# Accouchement de jumeaux conjoints de découverte fortuite au cours du travail au CHU de Dakar

**Published:** 2012-08-13

**Authors:** Mamour Guèye, Serigne Modou Kane Guèye, Mame Diarra Ndiaye Guèye, Abdoul Aziz Diouf, Mouhamadou Mansour Niang, Moussa Diallo, Mamadou Lamine Cissé, Jean Charles Moreau

**Affiliations:** 1 Clinique Gynécologique et Obstétricale, EPS Aristide Le Dantec, 1, Avenue Pasteur, BP 3001, Dakar, Sénégal

**Keywords:** Jumeaux conjoints, diprosopes, dicéphales, thoraco-omphalopages, conjoined twins, diprosopus, dicephalic, thoraco-omphalopagus

## Abstract

L'objectif de cette étude était de rapporter 3 cas de jumeaux conjoints, discuter de l'importance du diagnostic anténatal et de décrire les particularités diagnostiques, thérapeutiques et évolutives. Sur 45700 accouchements du 1er Février 2009 au 31 Décembre 2011, 3 cas de jumeaux conjoints ont été enregistrés, soit 1 cas pour 15000 accouchements. Ces cas ont été diagnostiqués au cours du travail au décours d'une dystocie mécanique ou d'une césarienne réalisée pour une autre indication. Il s'agissait d'un cas de jumeaux conjoints thoraco-omphalopages, un cas de diprosopes et un cas de dicéphales. L'accouchement dans les trois cas était fait par voie haute permettant d'extraire des mort-nés frais. Nous insistons sur l'intérêt d'un diagnostic anténatal précoce par le recours à l’échographie afin d’éviter les accidents mécaniques d'un accouchement qui ne saurait s'accomplir par voie basse.

## Introduction

Les jumeaux conjoints sont l'une des plus rares anomalies congénitales et l'un des plus grands challenges de la chirurgie pédiatrique [1]. L'accouchement à terme par voie basse des jumeaux conjoints est un événement rare pouvant mettre en jeu le pronostic maternel [2]. Aristote décrivit les premiers cas de jumeaux conjoints dans l'antiquité [3]. Depuis, plusieurs cas ont été rapportés mais les diagnostics étaient essentiellement faits à l'accouchement. Aujourd'hui, l’échographie peut diagnostiquer ce phénomène d'exception au premier trimestre de la grossesse et préciser le site d'union et les malformations fréquemment associées [3]. Le recours à l’échographie devrait être la règle, mais dans les pays en développement oö la médicalisation est encore insuffisante, les praticiens reçoivent régulièrement des parturientes sans suivi prénatal ou n'ayant bénéficié d'aucune échographie au cours de la grossesse. Nous rapportons 3 cas de jumeaux conjoints diagnostiqués au cours du travail devant une dystocie de dégagement ou au décours d'une césarienne réalisée pour une autre raison.

## Observations

### Observation 1: Jumeaux conjoints dicéphales (Figure 1: A et B)

Mme MSS, sixième geste, cinquième pare, âgée de 35 ans est admise en salle de travail pour un accouchement d'une grossesse estimée à terme. Elle a bénéficié de 2 consultations prénatales sans bilan biologique, ni échographie obstétricale. A l'admission, l'examen somatique était sans particularité, la hauteur utérine était de 36 cm, les bruits du cœur fœtal étaient perçus et réguliers, le col de l'utérus était dilaté à 8 cm, les membranes rompues, une présentation céphalique fixée. Une surveillance de l'accouchement a été décidée. La dilatation s'est complétée au bout de 2 heures suivie d'un début d'expulsion de la tête. Le dégagement s'est vite arrêté et la tête restait collée à la vulve évoquant une dystocie des épaules. Après une tentative de manœuvres pour résoudre une dystocie des épaules qui se sont soldées par un échec, une réévaluation précise de la situation a permis de percevoir un deuxième pôle céphalique dans l'utérus et de poser le diagnostic de jumeaux conjoints. Une embryotomie rachidienne pratiquée sur J1 est suivie d'une césarienne segmento-corporéale ayant permis d'extraire des jumeaux conjoints mort-nés frais, de sexe féminin pesant 3700 g. Il s'agissait de jumeaux conjoints dicéphales avec 2 têtes, un corps, un cordon ombilical comportant deux artères et une veine à la coupe. Les suites opératoires étaient simples, la patiente est sortie de l'hôpital à J4 post opératoire après blocage de la lactation.

**Figure 1:**
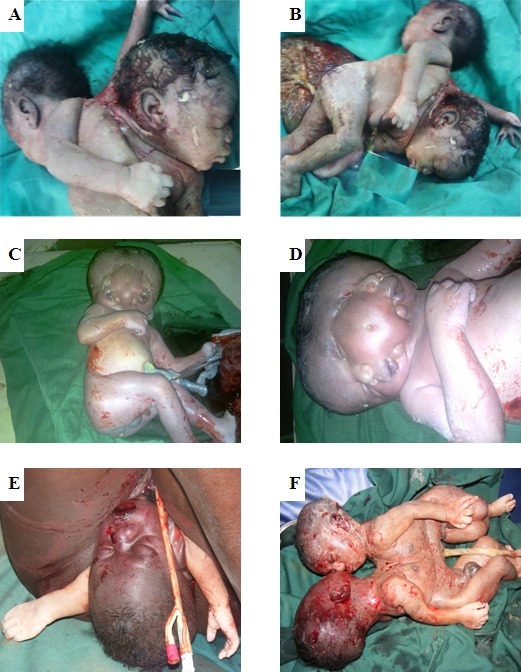
(A) Observation 1: Jumeaux conjoints dicéphales montrant après suture. (B): Observation 1: Jumeaux conjoints dicéphales montrant les trois membres supérieurs. (C): Observation 2: Jumeaux conjoins diprosopes (vue d'ensemble). (D): Observation 2: Jumeaux conjoins diprosopes (focus sur la tête). (E): Observation 3: Tête collée à la vulve avec procidence du cordon ombilical et de deux mains asymétriques. (F): Observation 3: Jumeaux conjoints thoraco-omphalopages après extraction

### Observation 2: Jumeaux conjoints diprosopes (Figure 1: C et D)

Mme AN, quatrième geste, quatrième pare, âgée de 36 ans était admise en salle de travail pour douleurs abdomino-pelviennes et métrorragies noirâtres sur une grossesse estimée à terme. Elle avait bénéficié de 2 consultations prénatales dans un poste de santé sans bilan biologique ni échographie obstétricale. A l'admission, la hauteur utérine était de 44 cm, l'utérus était tonique, sans relâchement interphasaire. Les bruits du cœur du fœtus n’étaient pas perçus au sthétoscope de Pinard. L'examen vaginal mettait en évidence un col utérin raccourci, admettant le doigt, les membranes étaient tendues. Ce tableau clinique était évocateur d'un hématome rétroplacentaire et avait motivé une rupture artificielle des membranes (le liquide amniotique était hématique) suivie d'une césarienne en urgence. La césarienne avait permis d'extraire des jumeaux conjoints diprosopes, de sexe masculin, morts-nés frais pesant 4250 g. Le placenta largement décollé pesait 800g et comportait une empreinte des caillots. Le poids de ces derniers était de 600 g. Les suites opératoires ont été simples, la patiente est sortie de l'hôpital à J5 post opératoire après blocage de la lactation.

### Observation 3: Jumeaux conjoints thoraco-omphalopages (Figure 1: E et F)

Mme FKC, deuxième geste de 18 ans, 1 enfant vivant, était évacuée d'un centre de santé pour suspicion de jumeaux conjoints sur une grossesse à terme en phase d'expulsion du travail. Elle n'avait pas d'antécédents familiaux de gémellité ni de consanguinité avec son conjoint. La grossesse était suivie avec 5 consultations prénatales et une échographie obstétricale ayant diagnostiqué la grossesse gémellaire sans plus d'informations. A l'admission, l'examen clinique retrouvait un bon état général, des muqueuses colorées. La hauteur utérine était de 42 cm. La palpation de la région sus pubienne était imprécise à cause d'une contracture utérine généralisée. Les bruits du cœur fœtal n’étaient pas perçus. L'examen vulvo-périnéal mettait en évidence un pôle céphalique accolé à la vulve associé à la procidence du cordon ombilical et de deux mains non symétriques. Ce tableau évoquait le diagnostic de jumeaux conjoints. L’échographie réalisée en salle de travail avait objectivé un deuxième pôle céphalique au dessus du détroit supérieur. Une laparotomie en urgence en vue d'une césarienne segmento-corporéale a permis d'extraire des jumeaux conjoints mort-nés frais thoraco-omphalopages, de sexe masculin, pesant 4200 g. Le placenta pesait 800g. L'incision utérine s'est propagée au vagin. La suture a pu être faite sans trop de difficultés. Les suites opératoires ont été simples, la patiente a pu sortir de l'hôpital à J4 post opératoire après blocage de la lactation.

## Discussion

La fréquence de survenue des jumeaux conjoints est de 0.2 pour 10.000 grossesses, et 0,05 pour 10.000 naissances vivantes [1]. L’étiopathogénie des jumeaux conjoints est mal connue. Il n'y a pas d'anomalie chromosomique associée. La race, l'hérédité, la parité et la consanguinité n'interviendraient pas dans le processus. Deux théories explicatives se dégagent [4]: la théorie de la fusion est incriminée dans la genèse de certaines formes, mais cette hypothèse est écartée au profit de la théorie de la scission. A partir du 9ème jour après la fécondation, les cellules de la lignée destinée à la formation du chorion et de l′amnios sont déjà différenciées, tout clivage de l’œuf survenant à partir de ce moment aboutira à une grossesse gémellaire monochoriale, monoamniotique Au-delà du quatorzième jour, la division est incomplète et aboutit à un monstre double ou jumeaux conjoints.

L'une des plus anciennes classifications date de 1573 et revient à Ambroise Paré [4]. En 1832, Saint-Hilaire établit une classification des jumeaux conjoints selon le site d'union externe et selon la symétrie [3]. Les jumeaux conjoints sont décrits par un ensemble d′adjectifs se terminant par le suffixe ‘pages’ (du mot grec pagos signifiant ce qui est fixé). De nombreux jumeaux ne correspondent pas parfaitement à ces classifications, et les termes sont souvent combinés pour décrire ces formes particulières. A cette classification, il faut ajouter les duplications incomplètes et les formes rares: les diprosopes (un tronc, une tête et deux faces), les dicéphales (un tronc et deux têtes), les dipygus (une tête, un thorax, un abdomen et deux pelvis), les jumeaux parasites (formes asymétriques, l'un des jumeaux est plus petit, moins formé et dépendant de l'autre), et le fœtus in fœtus (situation dans laquelle un fœtus imparfait est complètement inclus dans le corps du deuxième fœtus). Les thoraco-omphalopages représentent 70% des jumeaux conjoints [5]. Les diprosopes représentent l'entité la plus rare. Il existe une nuance entre les jumeaux conjoints Janiceps et les diprosopes. Dans le premier cas, les faces regardent dans des directions opposées alors que dans le deuxième, les faces sont reliées latéralement. Dans les deux cas, il s'agit d'une duplication des structures céphaliques, cette duplication pouvant être partielle ou complète [6]. Les jumeaux conjoints dicéphales représentent 11% des jumeaux conjoints et peuvent comporter 2, 3 ou 4 membres supérieurs. Ils sont morts-nés dans la plupart des cas à cause de l'existence de malformations cardiaques et pulmonaires. Mais il est clair aujourd'hui que les dicéphales comportant 3 ou 4 membres supérieurs peuvent avoir une survie prolongée, c'est le cas des frères écossais Tocci et Hensel et Rita et Christina [7].

En 1967, Rudolph rapportait le diagnostic par la radiographie du contenu utérin de 16 cas de jumeaux conjoints [3]. Wilson, en 1976 pose le premier diagnostic de jumeaux conjoints au cours d'une échographie réalisée sur une grossesse de 35 semaines [8]. Par la suite, les diagnostics sont faits de plus en plus précocement; même à sept semaines [3]. Même si le diagnostic échographique peut être réalisé précocement, il doit être toujours confirmé au cours d'une échographie de 12-13 SA. Aux deuxième et troisième trimestres, le diagnostic est plus facile, les organes étant plus développés [4].

A l’ère de l’échographie, aucun diagnostic de jumeaux conjoints ne devrait être une surprise de l'accouchement, même dans les pays en développement. Aujourd'hui, avec les efforts réalisés dans l'amélioration de l'accès aux soins au Sénégal, 9 femmes sur 10 bénéficient d'au moins une consultation prénatale au cours de la grossesse [9]. Au cours des consultations, l'attention du praticien devra être attirée par toute hauteur utérine excessive, la grossesse gémellaire doit être la hantise. Une échographie est souvent prescrite mais pas toujours réalisée par un personnel qualifié. Une enquête récente sur les connaissances, attitudes et pratiques de l’échographie obstétricale au Sénégal [10], a révélé que 66,6% des praticiens (médecins et sages-femmes) n’étaient pas diplômés en échographie. Ils étaient formés sur le tas et présentaient d'importantes lacunes en termes de connaissance et de pratique de l’échographie. En cas de grossesse gémellaire, le compte rendu doit être précis et étayé d'une iconographie claire. En effet, on notait sur le compte rendu d’échographie d'une de nos patientes (observation 3) que le diagnostic de grossesse gémellaire était sans plus de précision. Si le diagnostic avait été correctement réalisé, la conduite à tenir aurait été différente.

## Conclusion

Les jumeaux conjoints constituent une anomalie congénitale rare. Un diagnostic échographique précoce permet de mieux les cerner et de faire un bilan complet des malformations. L'accouchement dans un milieu obstétrico-chirurgical et néonatal permet de mieux prendre en charge les nouveaux-nés et d’éviter les dystocies. Dans les pays en développement, toutes les femmes devraient bénéficier d'au moins une échographie obstétricale morphologique réalisée par un personnel qualifié.

## References

[1] Agarwal K, Agarwal L, Agrawal VK, Agarwal A (2011). Conjoined Twins: A Report of 3 Cases to Emphasize Prenatal Diagnosis and Challenges. NJOG..

[2] N'Dinga GH, Iloki HL (2011). Dystocic expulsion of thoracopagus conjoined twins in the Talangai hospital, Republic of Congo. Pan Afr Med J..

[3] Cuillier F, Lemaire P, Sommer JC, Abossolo T (2001). Découverte anténatale de jumeaux conjoints omphalopages à 13 semaines d'aménorrhée. Gynecol Obstet Fertil..

[4] Broussin B (2000). Les jumeaux conjoints: diagnostic anténatal. J Pediatr Puericulture..

[5] Spitz L (2005). Conjoined twins. Prenat Diagn..

[6] D'Armieto M, Falleti J, Maruotti GM, Martinelli P (2010). Diprosopus conjoined twins: Radiologic, autoptic, and histologic study of a case. Fetal and Pediatric Pathology..

[7] Bondeson J (2001). Dicephalus Conjoined Twins: A Historical Review With Emphasis on Viability. J Pediatr Surg..

[8] Wilson RL, Cetrulo CL, Shaub MS (1976). The prepartum diagnosis of conjoined twins by the use of diagnosis ultrasound. Am J Obstet Gynecol..

[10] Sarr OJD (2011). Pratique de l’échographie dans les villes de Dakar et Thiès (Sénégal). Thèse Méd, Dakar.

